# A Peptide Motif Covering Splice Site B in Neuroligin-1 Binds to Aβ and Acts as a Neprilysin Inhibitor

**DOI:** 10.1007/s12035-024-04475-z

**Published:** 2024-09-11

**Authors:** Lene T. Dietz, Katrin Põld, Balázs A. Györffy, Alexander Zharkovsky, Jakob B. Sørensen, Stanislava Pankratova, Oksana Dmytriyeva

**Affiliations:** 1 Department of Neuroscience, Faculty of Health and Medical Sciences, University of Copenhagen, Copenhagen, Denmark; 2 Department of Pharmacology, University of Tartu, Tartu, Estonia; 3 Research Laboratory for Stereology and Neuroscience, Bispebjerg-Frederiksberg Hospital, University Hospital of Copenhagen, Copenhagen, Denmark; 4 Section of Comparative Pediatrics and Nutrition, Faculty of Health and Medical Sciences, University of Copenhagen, Copenhagen, Denmark; 5 Novo Nordisk Foundation Center for Basic Metabolic Research, Faculty of Health and Medical Sciences, University of Copenhagen, Blegdamsvej 3, 2100 Copenhagen, Denmark

**Keywords:** Neuroligin-1, Amyloid-β, Neprilysin, Alzheimer’s disease

## Abstract

The most common cause of dementia among elderly people is Alzheimer’s disease (AD). The typical symptom of AD is the decline of cognitive abilities, which is caused by loss of synaptic function. Amyloid-β (Aβ) oligomers play a significant role in the development of this synaptic dysfunction. Neuroligin-(NL)1 is a postsynaptic cell-adhesion molecule located in excitatory synapses and involved in the maintenance and modulation of synaptic contacts. A recent study has found that Aβ interacts with the soluble N-terminal fragment of NL1. The present study aimed to elucidate the role of NL1 in Aβ-induced neuropathology. Employing surface plasmon resonance and competitive ELISA, we confirmed the high-affinity binding of NL1 to the Aβ peptide. We also identified a sequence motif representing the NL1-binding site for the Aβ peptide and showed that a synthetic peptide modeled after this motif, termed neurolide, binds to the Aβ peptide with high affinity, comparable to the NL1-Aβ interaction. To assess the effect of neurolide in vivo, wild-type and 5XFAD mice were subcutaneously treated with this peptide for 10 weeks. We observed an increase in Aβ plaque formation in the cortex of neurolide-treated 5XFAD mice. Furthermore, we showed that neurolide reduces the activity of neprilysin, the predominant Aβ-degrading enzyme in the brain. Accordingly, we suggest that neurolide is the NL1-binding site for Aβ peptide, and acts as an inhibitor of neprilysin activity. Based on these data, we confirm the involvement of NL1 in the development of AD and suggest a mechanism for NL1-induced Aβ plaque formation.

**Supplementary Information:**

The online version contains supplementary material available at 10.1007/s12035-024-04475-z.

## Introduction

One of the major neuropathological hallmarks of progressed Alzheimer’s disease (AD) is the deposition of extracellular plaques composed mainly by 40–42 amino acid amyloid-β peptides (Aβ). In addition, Aβ oligomers target synaptic complexes, causing synapse dysfunction, and this process is associated with memory decline [1–3]. Beta-amyloid peptides result from the proteolytic cleavage of the amyloid precursor protein (APP). The amyloidogenic proteolytic pathway includes the activation of two enzymatic systems of β- and γ-secretases. Mutations in APP and/or components of the γ-secretase complex, including presenilins, are the main cause of early-onset familial AD (FAD) [4]. Two enzymes, the metalloproteases neprilysin (NEP) and insulin-degrading enzyme (IDE), can effectively degrade Aβ peptides [5]. A recent study has shown that NEP deficiency contributes to neuropathological and behavioral phenotypes in a transgenic mouse model of AD. The AD rodent model, 5XFAD mice, shows strongly decreased levels of NEP expression compared to wild-type mice [6]. Thus, NEP deficiency could be one of the factors that accelerate Aβ accumulation and plaque deposition.

Neuroligins (NLs) are synaptic adhesion molecules involved in synaptic maturation and functioning. Three neuroligin-encoding genes (*NL1-3*) are mostly expressed in the central nervous system in mice and rats. Humans additionally express *NL4* and *NL4Y* [7, 8]. NL1 is usually expressed in neurons at excitatory postsynaptic sites and found bound to NMDA receptors, postsynaptic dencity-95 (PSD-95), synaptic scaffolding molecule (S-SCAM), Thrombospondin 1, γ-Protocadherins, and MAM Domain Containing Glycosylphosphatidylinositol Anchor 1 (MDGA1) protein [9–15]. All NLs have one conserved alternative splice site (A). However, NL1 is unique among other NLs in that its gene has an additional alternative splice insert B (NL1(+ B)) in the extracellular domain, which contains an N-glycosylation consensus sequence at Asn303 [16] and specifically contributes to the binding strength to its binding partner, neurexin NX1β [8, 17].

Synaptic activity is associated with acute cleavage of NL1 from postsynaptic terminals by MMP9 [18] and by disintegrin and metalloproteinase domain-containing protein 10 (ADAM10 [19]). The cleavage of the extracellular domain of NL1 weakens the synapse by rapidly decreasing presynaptic transmitter release [19], most likely via activation of metabotropic glutamate receptor 2 (mGluR2), followed by a decrease in cyclic adenosine monophosphate (cAMP) formation [20]. Furthermore, the NL1 ectodomain directly binds to Aβ, thereby promoting the formation of toxic Aβ oligomers [21]. Thus, NL1 might play a role in synaptic dysfunction in AD.

We have recently shown that a synthetic peptide, neurolide, modeled after part of the binding site of NL1 to NX1β, mimics biological properties of the NL1 ectodomain [22], stimulates phosphorylation of the NMDA receptor subunit in vitro, and rescues an MK-801-induced decrease in long-term potentiation (LTP) and memory impairment [23]. Here, we show that the NL1 ectodomain and the neurolide peptide both strongly interact with Aβ (K_D_ is approximately 20 nM). Neurolide dose-dependently inhibits NEP activity in vitro. Systemic treatment of 5XFAD mice with neurolide led to a marked increase in Aβ-containing plaque deposition in the cortex, thus suggesting that soluble NL1 contributes to Aβ deposition by inhibiting NEP activity.

## Material and Methods

### Proteins and Peptides

Recombinant rat NL1 and NX1β were purchased from R&D systems (Abingdon, UK). Nine NL1-derived peptides, including previously described neurolide, SEGNRWSNSTKGLFQRA [22], and eight new motifs, NLp1-8, SGGPKPVMYIHG, ITVNYRLG, ASFQPAKYA, AENIVDSDDG, ASDFDFAVSN, AHTKPNRFEA, SVPVTSAFPTAKQDDA, and ATADLHSNFGS (according to UniProtKB/Swiss-Prot Q62765) were synthesized as tetramers coupled to lysine backbone using the solid-phase Fmoc protection strategy (Schafer-N, Copenhagen, Denmark). Purity was estimated at ≥ 80% by high-performance liquid chromatography. The peptides were reconstituted in sterile water, and the concentrations were determined by measuring absorbance at 205 nm. Aβ_1-42_, Aβ_1-40_, and biotinylated Aβ_1-40_ and Aβ_40-1_ were purchased from Bachem AG (Bubendorf, Switzerland) or Schafer-N, and dissolved in dimethyl sulfoxide (DMSO). The sequential alignments of NL1 sequence with Aβ_25-35_ were done using the Clustal Omega online service [24].

### Surface Plasmon Resonance (SPR) Analysis

Binding analysis was performed on a Biacore 2000 SPR biosensor (GE Healthcare) at 25 °C using HBS-P (10 mM HEPES, pH 7.4, 150 mM NaCl, 0.005% [v/v] Surfactant P20) supplemented with 3 mM CaCl_2_ as a running buffer. Approximately 2000 Resonance Units (RU) of NL1 was immobilized on a CM4 sensor chip at a flow rate of 5 μl/min by amine coupling. Binding to serially diluted recombinant NX1β, used as a positive control, was performed as previously described [22]. Aβ_1-42_ was dissolved in anhydrous DMSO to a stock concentration of 3.21 mM followed by serial dilution in running buffer. Peptide was injected into the chip, and binding experiments were performed at a flow rate of 20 μl/min, followed by regeneration of sensor chip with 10 μl of 15 mM NaOH and 150 mM NaCl. After subtraction of blank control, the binding curves were analyzed based on a nonlinear curve fitting using a 1:1 Langmuir binding model and the software package BIAevaluation 4 (GE Healthcare). The apparent equilibrium constant (K_D_) was calculated as k_d_/k_a_, in which k_a_ and k_d_ are the apparent association and dissociation constants, respectively.

### Enzyme-Linked Immunosorbent Assay (ELISA)

NL1 and NL1-derived peptide binding to Aβ was measured by competitive ELISA using the shorter Aβ_1-40_ peptide with a slower polymerization constant, optimized for this binding assay [21]. Recombinant NL1 (3.125 nM) was immobilized on a poly-sorb 96-well plate (NUNC, Roskilde, Denmark) using sodium bicarbonate buffer (pH 9.6), and left overnight at 4 °C, followed by washing with PBST buffer (PBS supplemented with 0.1% [vol/vol] Tween-20, pH 7.4). In separate tubes, biotinylated Aβ_1-40_, at final concentration 400 nM, was premixed with serially diluted NL1 or NL1-derived peptides for 30 min. A reversed version of Aβ (Aβ_40-1_) was used as the respective negative control (Supplementary Fig. 1). After preincubation, the samples were centrifuged at 20,000 × *g* for 10 min at 4 °C and 100 μl of the mixtures were transferred in triplicates onto the precoated ELISA plate. Plates were incubated at room temperature for 4 h, followed by washing and incubation with streptavidin-HRP conjugate (1:1000, 1 h; Dako, Glostrup, Denmark). The signal was developed with TMB ONE (KEM-EN-TEC, Denmark), and the reaction was terminated with 0.2 M H_2_SO_4_. Absorbance was measured at 450 nm on a Wallac VICTOR2 1420 Multilabel Counter (PerkinElmer, Hvidovre, Denmark). K_D_ values were derived by sigmoidal dose–response (variable slope) fit of NL1 and neurolide dose–response curves after log transformation according to GraphPad Prism (version 6, GraphPad, San Diego, CA, USA). Obtained inhibitory concentration IC50 value was interpreted as K_D_ value.

### Aggregation Assay and Immunoblot Validation

Aβ_1-42_ was dissolved in sterile deionized water to a final concentration of 1 mM. Then, the peptide was subjected to a basic shock by adding 2 M NaOH to pH 12 followed by neutralization with 1 M HCl. Aβ_1-42_, at a final concentration of 75 μM (pH 7.4) either alone or in combination with NL1 (1 μM), neurolide (10 μM), or NLp7 (10 μM), was loaded in triplicates on 96-well plate. Immediately after mixing the samples, thioflavin T (5 μM, ThT; Sigma-Aldrich), dissolved in the aggregation buffer (50 mM Tris, pH 7.4), was added to wells and plate was incubated at 37 °C for 8 days. The formation of beta-amyloid aggregates was monitored first for 210 min and then every 24 h by recording changes in ThT fluorescence at excitation/emission (Ex/Em) = 440/482 nm.

To verify that the protein and peptide remained intact after the prolonged incubation, the His-tagged and purified soluble ectodomain of NL1 (sNL1) and biotinylated neurolide (b-neurolide) were prepared as above. After 8-day incubation, they were subjected to conventional SDS polyacrylamide gel electrophoresis on a pre-casted gel (Any kD Mini-PROTEAN TGX gel, Bio-Rad) and transferred to a PVDF membranes (Millipore) together with freshly prepared samples of sNL1 and b-neurolide as corresponding positive controls. Subsequently, membranes were blocked in 5% (wt/vol) non-fat dry milk in PBST (PBS containing 0.05% [vol/vol] Tween-20). To detect sNL1, blots were probed overnight with a 6x-His tag monoclonal antibody (1:2000, Thermo Fisher Scientific) at 4 °C, followed by incubation for 1 h at room temperature with an anti-mouse horseradish peroxidase (HRP)-conjugated secondary antibody (1:10,000, Thermo Fisher Scientific). B-neurolide was detected by incubation for 1 h at room temperature with a streptavidin-HRP complex (1:5000, Dako). Chemiluminescent signal on the blot was developed by employing the SuperSignal ELISA Femto Substrate kit (Thermo Fisher Scientific) according to the manufacturer’s instructions, and images were acquired using an AlphaImager system (Alpha Innotech).

### Determination of Neprilysin (NEP) Activity

#### Screening of Compounds Using Recombinant Enzyme

NEP activity was measured using the SensoLyte 520 Neprilysin Activity Assay Kit *Fluorimetric* (Anaspec, Fremont, California, USA) following the protocol provided by the manufacturer. Briefly, Aβ_1-42_ was dissolved in anhydrous DMSO to a stock concentration of 5 mM, followed by dilutions in assay buffer (100 μM, 50 μM, and 5 μM). Both NL1 (3 μM, 1 μM, 0.3 μM, 0.1 μM) and neurolide (100, 10, 5, and 1 μM) were dissolved in assay buffer. Randomly selected two additional NL1-derived peptides, NLp4, and NLp7 (5 μM, Table 1), were included in the analysis. The NEP solution (40 μl) was mixed with 10 μl of either NL1 or peptides and supplemented with 50 μl of NEP substrate (5-FAM/QXL), and the final mixture was incubated in black optical-bottom 96-well plate (Nunc, Rochester, New York, USA). In co-stimulation experiments, Aβ_1-42_, prepared as described above, was added to the solution in final concentrations either 0.5 μM or 5 μM. The fluorescence signal was recorded at Ex/Em = 485/535 nm every 20 min for 2 h. The data are presented as the relative activity of NEP alone.

**Table 1:** Sequences of NL1-derived peptides and their binding affinities to Aβ_1-42_

Peptide name	Sequence	*K*_D_
Neurolide [22]	SEGNRWSNSTKGLFQRA	35.26 ± 2.3 nM
NLp1	SGGPKPVMYIHG	No binding
NLp2	ITVNYRLG	675.7 ± 35.67 nM
NLp3	ASFQPAKYA	No binding
NLp4	AENIVDSDDG	No binding
NLp5	ASDFDFAVSN	No binding
NLp6	AHTKPNRFEA	No binding
NLp7	SVPVTSAFPTAKQDDA	No binding
NLp8	ATADLHSNFGS	No binding

*K*_D_, equilibrium dissociation constant

#### Screening of Compounds Using NEP-Enriched Brain Membrane Fraction

To measure NEP activity in brain tissue, whole brains without cerebellum were dissected from adult mice (C57Bl/6j or 5XFAD) and homogenized with a Teflon-glass homogenizer in five volumes (w/v) of ice-cold lysis buffer containing PBS (pH 7.4), 0.25 M sucrose and protease inhibitor cocktail (Complete™, EDTA-free, Roche Diagnostics, Indianapolis, IN). The homogenates were centrifuged at 9000 × *g* for 15 min at 4 °C. The supernatants were further centrifuged at 20,000 × *g* at 4 °C for 20 min. The resulting pellets were solubilized in resuspension buffer containing PBS (pH 7.4) and 1% Triton X-100 for 1 h on ice and again centrifuged at 20,000 × *g* at 4 °C for 20 min. Clear supernatants were used as membrane-extracted fractions. Protein concentrations of Triton-extracted membrane fractions were determined using the Pierce BCA Protein Assay kit (Thermo Fisher Scientific). The standard assay mixture consisted of membrane fraction containing 40 μg total protein diluted in 40 μl of dilution buffer, 10 μl of testing compound (NL1, neurolide or vehicle), and 50 μl of NEP substrate (5-FAM/QXL; Anaspec) in a total volume of 100 μl. The reaction was initiated by adding substrate to the assay mixture. To determine the specificity of the assay, a set of samples were incubated with 10 μl of thiorphan (100 nM final concentration), a NEP-specific inhibitor (Anaspec) [5, 25]. Positive and negative controls were set up according to the manufacturer’s protocol (Anaspec). Fluorescence was measured at Ex/Em = 485/535 nm every 20 min for 2 h. The data were expressed as percentage of relative activity of neprilysin.

### Animal Experiments

All animal experiments were performed in accordance to European Union law with licenses from the Danish Animal Experiments Inspectorate and from the Ethical Committee of the Estonian Ministry of Agriculture. The number of animals utilized in the experiment was kept to a minimum, and all work was conducted in a way to cause as little harm and suffering to the animals as possible. Transgenic 5XFAD mice on C57Bl/6j × SJL genetic background were purchased from Jackson Laboratories (strain: B6SJL-Tg (APPSwFlLon, PSEN1*M146L*L286V) 6799Vas/J) and backcrossed to C57Bl/6j wild-type mice. 5XFAD transgenic mice overexpress mutant human APP(695) with the Swedish (K670N and M671L), Florida (I716V), and London (V717I) Familial Alzheimer’s Disease (FAD) mutations along with human PS1 harboring two FAD mutations, M146L and L286V. The mice were genotyped by PCR analysis of tail DNA. Wild-type (WT) mice were littermates to 5XFAD.

At the age of 2 months, 5XFAD and WT mice were injected subcutaneously (s.c.) with either neurolide (10 mg/kg) or saline in a volume of 0.1 ml/10 g of body weight three times a week for 10 weeks.

### Y-maze

To estimate the effect of neurolide on spatial working memory, the Y-maze test was used, wherein the recording of alternation behavior was assessed. Mice were placed in the middle of the three-arm maze, joined with equal angle between all arms, and allowed to explore the maze freely for 8 min. The sequence and total number of entries into each arm were assessed in video recordings. Arm entry was considered completed when the hind paws of the mouse had been placed in the arm. An actual alternation was defined as entries into all three arms on consecutive choices. Percentage alternation was calculated as the ratio of actual to possible alternation (defined as total number of arm entries − 2) × 100, as follows: $\%\, \text{Alternation}=\left(\left(\text{Number of alternations}\right)/\left(\text{Total arm entries}-2\right)\right)\times 100.$ 

### Novel Object Recognition Task

The novel object recognition assay is based on the innate tendency of animals to differentially explore novel and familiar objects [26] and consists of habituation, training, short-term (2 h) and long-term (24 h) memory retention sessions. On day 1, the mice were habituated to the test area (a 35 × 35 cm box with a floor divided into 16 squares). Each mouse was placed into a box without objects and allowed to explore it freely for 5 min. During a training session, a mouse was placed in the test box and exposed to 2 identical nontransparent glass objects placed in the box. During 5 min, locomotion and exploration time for both objects were recorded. Exploration of an object was defined as pointing the nose to the object at a distance of ≤ 1 cm and/or touching it with the nose. Climbing or sitting on the object was not considered exploration [27]. Short-term memory retention was evaluated 2 h after the training session, whereas long-term memory retention was evaluated 24 h after the training session. During both sessions, mice were exposed to the familiar object and to a novel object. The exploration time for the familiar and novel objects and locomotion were recorded for 5 min. Mice failing to explore one or both objects in the training trial were excluded from the statistical analysis.

### Tissue Preparation and Immunohistochemistry

After 10 weeks of treatment, mice were transcardially perfused with 0.1 M PBS, pH 7.4 under deep pentobarbital anesthesia (intraperitoneal, 200 mg/kg). The left brain hemispheres were postfixed in 4% paraformaldehyde in PBS. The brain hemispheres were further dehydrated in ethanol, cleared, embedded in paraffin blocks and serially sectioned into 7-μm coronal sections through the entire brain hemisphere. Parallel series of sections were collected, consisting of 12 equally spaced Sects. (400 μm apart). One of the series was stained with 1% Thioflavin-S for histological evaluation of Aβ plaque formation [28]. Another series was used for immunohistochemical staining of GFAP + astrocytes as described previously [29]. Extracellular Aβ plaque load was assessed in cortex, hippocampus, striatum, thalamus, and hypothalamus. GFAP-positive astrocytes were assessed in motor cortex. Serial super images of whole-brain sections were acquired using an Olympus BX-51 microscope equipped with the Visiopharm Integrator System software (Visiopharm) and analyzed using the ImageJ software package. The pictures were converted to 8-bit images, and the area of interest was marked. The intensity threshold was fixed to fit Thioflavin-S or GFAP signal. The area, covered by Thioflavin-S or GFAP staining, was measured in selected regions.

### Statistical Analysis

Data were analyzed by one-sample Student’s *t*-test (neprilysin enzyme activity), lineal regression analysis (aggregation assay), two-way ANOVA followed by Dunnet’s post hoc test (Y-maze and novel object recognition test), unpaired (histology), or paired (Aβ aggregation assay) Student’s *t*-tests using GraphPad Prism, version 6 (GraphPad, San Diego, CA, USA). All values are expressed as the mean ± SEM. The levels of significance are denoted as follows: *p < 0.05, **p < 0.01, ***p < 0.001, and ****p < 0.0001. Values of *p* < 0.05 were considered statistically significant.

## Results

### Part of the NL1-NX1β Interaction Interface Is a NL1 Binding Site for Aβ

**Fig. 1:**
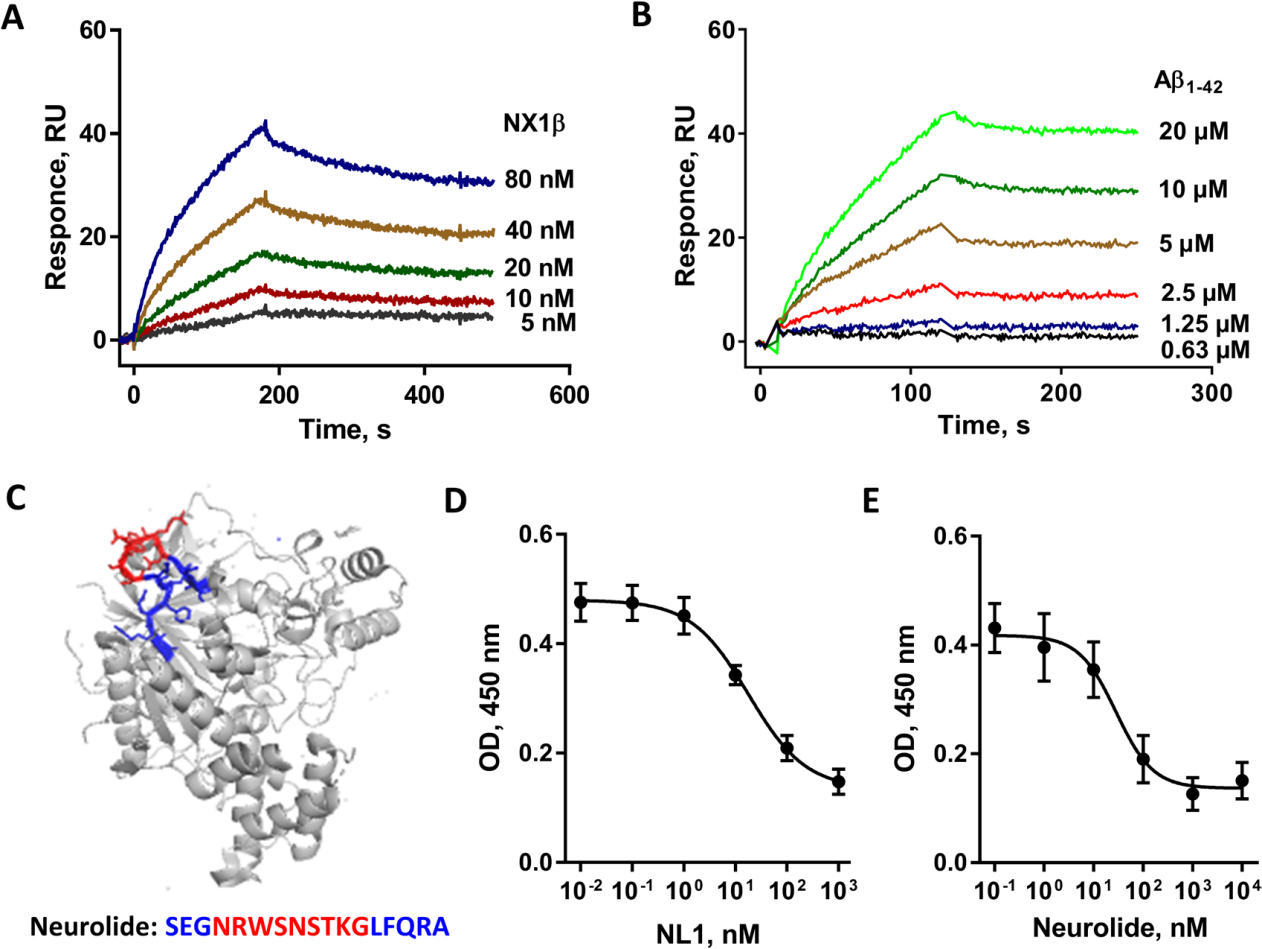
Both NL1 and NL1-derived peptide, neurolide, bind to Aβ. **A**, **B** For SPR binding experiments, recombinant NL1 was immobilized on a CM4 sensor chip and **A** positive control, NX1β, or **B** Aβ_1-42_ were injected into the chip at the indicated concentrations and at a flow rate of 20 μL/min. Sensorgrams of blank-subtracted responses representative from four independent experiments are shown. RU, resonance units. **C** Ribbon diagram of NL1 (gray) with neurolide motif highlighted in blue and red, wherein the splice insert **B** is depicted in red (PDB: 5OJK) [13]. **D**, **E** Results from competitive ELISA showed the binding of immobilized NL1 (**D**) or neurolide (**E**) to serially diluted Aβ_1-40_

The soluble ectodomain of NL1 (sNL1) binds to Aβ_1-40_ and Aβ_1-42_ [30]. To investigate the interaction between NL1 and Aβ in detail, we applied two approaches: surface plasmon resonance (SPR) and competitive ELISA. Using SPR, the extracellular domain of NL1 was immobilized on a sensor chip. To verify the binding capacity of NL1, a serially diluted positive control, Neurexin-1β (NX1β), was injected over the chip. The results showed that NX1β binds to immobilized NL1 with low nanomolar affinity (6.64 nM), confirming the proper folding and function of the immobilized protein (Fig. 1A). Furthermore, the results from the SPR analysis showed that Aβ_1-42_ binds to NL1 with a K_D_ value of 72 nM ± 24 nM, (Fig. 1B), thus confirming the previously shown nanomolar affinity of Aβ to NL1 [21]. Of note, the affinity constant K_D_ of NL1 to Aβ is approximately 11 times lower compared to NX1β. Competitive ELISA, an alternative approach, further demonstrated the binding between Aβ_1-40_ and NL1, with a K_D_ of 19.9 nM ± 7.6 nM (Fig. 1D). To identify the binding motif(s) within NL1 responsible for its interaction with Aβ, we analyzed the NL1 sequence to identify short motifs bearing similarity to Aβ_25-35_, the shortest fragment of Aβ_1-42_ with self-aggregation propensity (Supplementary Fig. 2). We delineated eight peptide sequences, NLp1-8 (Table 1), in addition to previously described neurolide peptide (Fig. 1C; [22]). Among all NL1-derived peptides, a high-affinity interaction was detected only between Aβ_1-40_ and neurolide, with a K_D_ of 35.3 ± 2.3 nM (Fig. 1E and Table 1). The reverse Aβ peptide, Aβ_40-1_, employed as a control, binds to neurolide with significantly lower affinity (K_D_ 7.5 μM; *p* < 0.0001), with signal enhancement detected solely at the highest tested concentration of neurolide, likely attributable to non-specific complex formation (Supplementary Fig. 1). Of the eight other NL1-derived peptides, only NLp2 showed weak binding to Aβ_1-40_, with a K_D_ value of 676 ± 36 nM, which is approximately 22 times lower than the affinity of neurolide to Aβ (*p* < 0.05). The other tested peptides showed no binding to Aβ (Table 1). Because the location of the NLp2 motif inside the tertiary structure of NL1 protects the sequence from exposure to the surrounding environment (Protein Data Bank (PDB) 5OJK, [13]), NLp2 was omitted from the list of Aβ-binding motifs. Altogether, our results suggest that NL1 binds to Aβ and that the binding motif overlaps with the binding motif for NX1β.

### Neurolide Attenuates Aβ Polymerization In Vitro

**Fig. 2:**
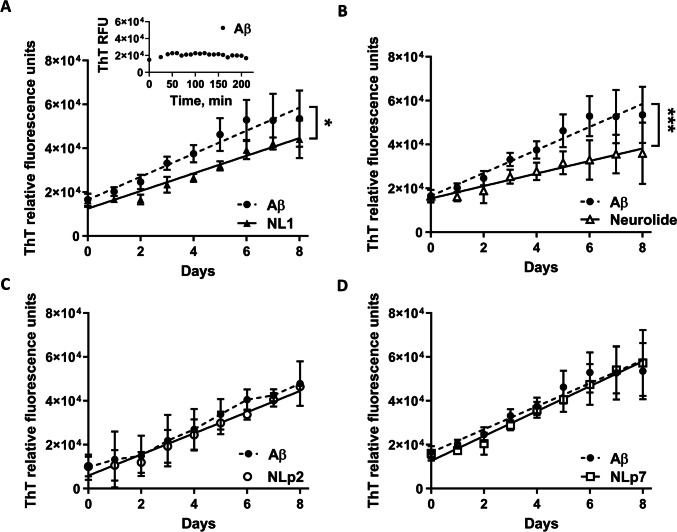
Both sNL-1 and neurolide modulate Aβ aggregation. The formation of Aβ amyloid aggregates was tracked by ThT fluorescence measurements either for 210 min (insert in (**A**)) or over 8 days, using 75 μM of Aβ_1-42_ alone or co-incubated with either 1 μM sNL1 (**A**) or 10 μM of neurolide (**B**). Treatment with NL1 and neurolide, but not with 10 μM of NLp2 or NLp7 (**C**, **D**), showed a significant decrease in the formation of aggregates in comparison with Aβ alone (dashed lines for NLp7 and NLp2). Slopes differences between lineal regression lines for Aβ and NL1, *F* = 0.033, *p* = 0.033; Aβ and neurolide, *F* = 26.34, *p* = 0.0002; and Aβ and NLp7, *F* = 0.4, *p* = 0.4

Previously, the soluble domain of NL1 has been shown to affect Aβ aggregation with a dual effect, depending on the Aβ:sNL1 ratio [21]. Since we showed that neurolide is an Aβ-binding motif within NL1, we investigated whether neurolide would affect Aβ aggregation. Incubation of the Aβ_1-42_ peptide alone (75 μM) for 8 days, rather than for a brief period of 210 min, led to the formation of Aβ amyloid aggregates, as reflected by the increase in ThT fluorescence over time (Fig. 2A, dashed line, and insert). While in the presence of high concentrations of sNL1 (1 μM), the rise of ThT fluorescence was significantly decreased since the slopes for the two regression lines were significantly different (*F* = 5.561, *p* = 0.033), thus confirming previous results obtained with both Aβ_1-40_ and Aβ_1-42_ [21]. The decrease in ThT fluorescence was also observed when Aβ_1-42_ was incubated with 10 μM neurolide (Fig. 2B); the difference between the slopes of these two regression lines was very significant (*F* = 26.34, *p* = 0.0002), confirming that neurolide was able to attenuate Aβ peptide aggregation in vitro. The observed decrease in ThT fluorescence was not due to potential degradation of NL1 and neurolide since the results of Western blot showed that both NL1 and neurolide remained intact after the prolonged incubation (Supplementary Fig. 3). Incubation of all other NL1-derived peptides, including NLp2 and Aβ–non binding peptide NLp7 (10 μM; Table 1), had no effect on the development of ThT fluorescence (Fig. 2C, D; slopes difference *F* = 0.76, *p* = 0.4 for NLp7), confirming the sequence-specific effect of neurolide on Aβ aggregation.

### The In Vivo Effect of Neurolide Is Dependent on Genotype

**Fig. 3:**
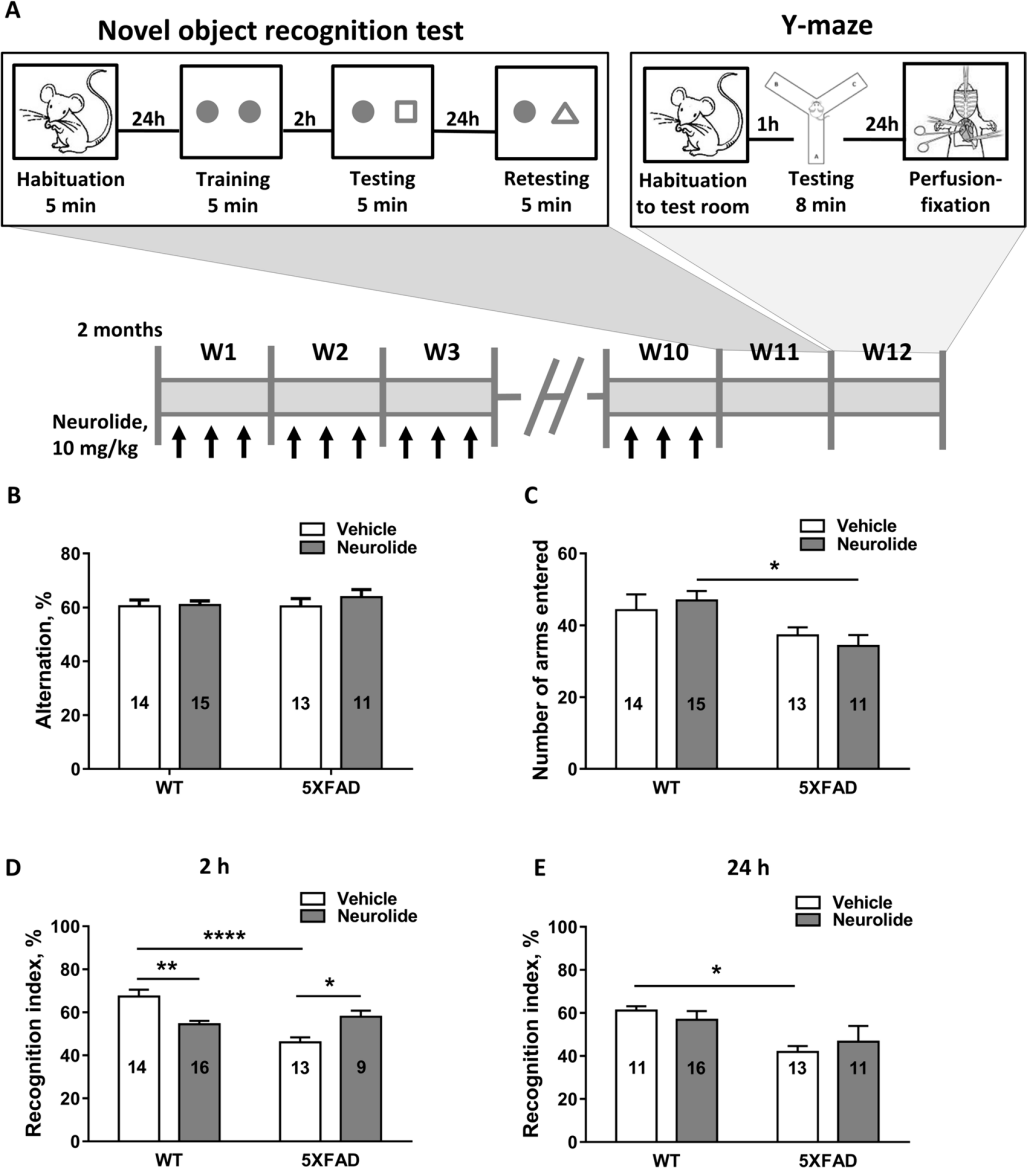
Effect of chronic administration of neurolide on memory in WT and 5XFAD mice. **A** Timeline of the in vivo experiments. Both WT and 5XFAD mice were s.c. injected (black arrow) with either vehicle or neurolide three times per week for 10 weeks (W) from the age of 2 months. **B** Y-maze test, performed at week 11, showed no neurolide effect on spontaneous alterations. **C** Number of arms entered either by WT or 5XFAD mice was not different within each genetic group; however, among neurolide-treated mice, 5XFAD transgene mice showed a significant decrease in the number of entrances. The neurolide effect on short-term (**D**) and long-term (**E**) memory was estimated using the novel object recognition test. The recognition index was calculated as 100 × Tn/(Tf + Tn), wherein Tf and Tn are the time spent on investigating a familiar and novel object, respectively. Number of animals is indicated in columns. All results are expressed as the mean ± SEM

Because neurolide was capable of binding Aβ with high affinity, we hypothesized that it could diminish the formation of Aβ plaques by offering additional binding sites for Aβ monomers. Neurolide can pass the blood–brain-barrier in the range of nanograms [23] and thus may be present in brain parenchyma. Therefore, we investigated the effect of s.c. administered neurolide on mouse behavior and the formation of plaques in WT and 5XFAD mice (Fig. 3A). The 5XFAD mice generate almost exclusively Aβ_1-42_—the most toxic Aβ species—at high levels. We administered neurolide to mice starting at 2 months of age, when cerebral Aβ deposition begins and continued, for the following 12 weeks, when cognitive deficits are observed (4–6 months of age), as it was previously described for this transgenic AD model [31].

#### Y-maze

We investigated the effect of neurolide on spatial memory in 5-month-old WT and 5XFAD mice employing the Y-maze test. In contrast to previously published findings [31], we found no significant difference in Y-maze alternation rate between WT mice and 5XFAD littermates (Fig. 3B), no significant interaction between group and neurolide treatment. Conversely, 5XFAD mice showed lower locomotion and exploratory activity than WT littermates, as manifested in the decreased number of arms entered (two-way ANOVA, *p*-value for interaction 0.3944; *p*-value for genotype 0.0037) (Fig. 3C). Neurolide treatment had no effect on alternation rate or on the number of arms entered (Fig. 3B, C) (*p*-value for treatment 0.9532). Neurolide induced no improvement in spatial memory in WT or 5XFAD mice.

#### Novel Object Recognition

We also investigated whether neurolide counteracts the effect of cognitive decline in 5XFAD mice by employing the object recognition test. We detected a statistically significant decrease in short- (2 h, *p* < 0.0001) and long-term (24 h, *p* < 0.05) memory in 5XFAD animals in comparison with WT littermates (Fig. 3D, E). In the 5XFAD group, neurolide treatment significantly enhanced the recognition index (*p* < 0.05), whereas vehicle-treated animals had a significantly higher recognition index than neurolide-treated mice (*p* < 0.01) in the WT group when testing short-term memory (Fig. 3D). The same tendency was observed after 24 h; however, the differences were no longer statistically significant (Fig. 3E). Based on these results, we conclude that neurolide improves cognitive memory in 5XFAD mice but impairs memory in WT mice.

### Neurolide Increases Plaque Formation in 5XFAD Mice

**Fig. 4:**
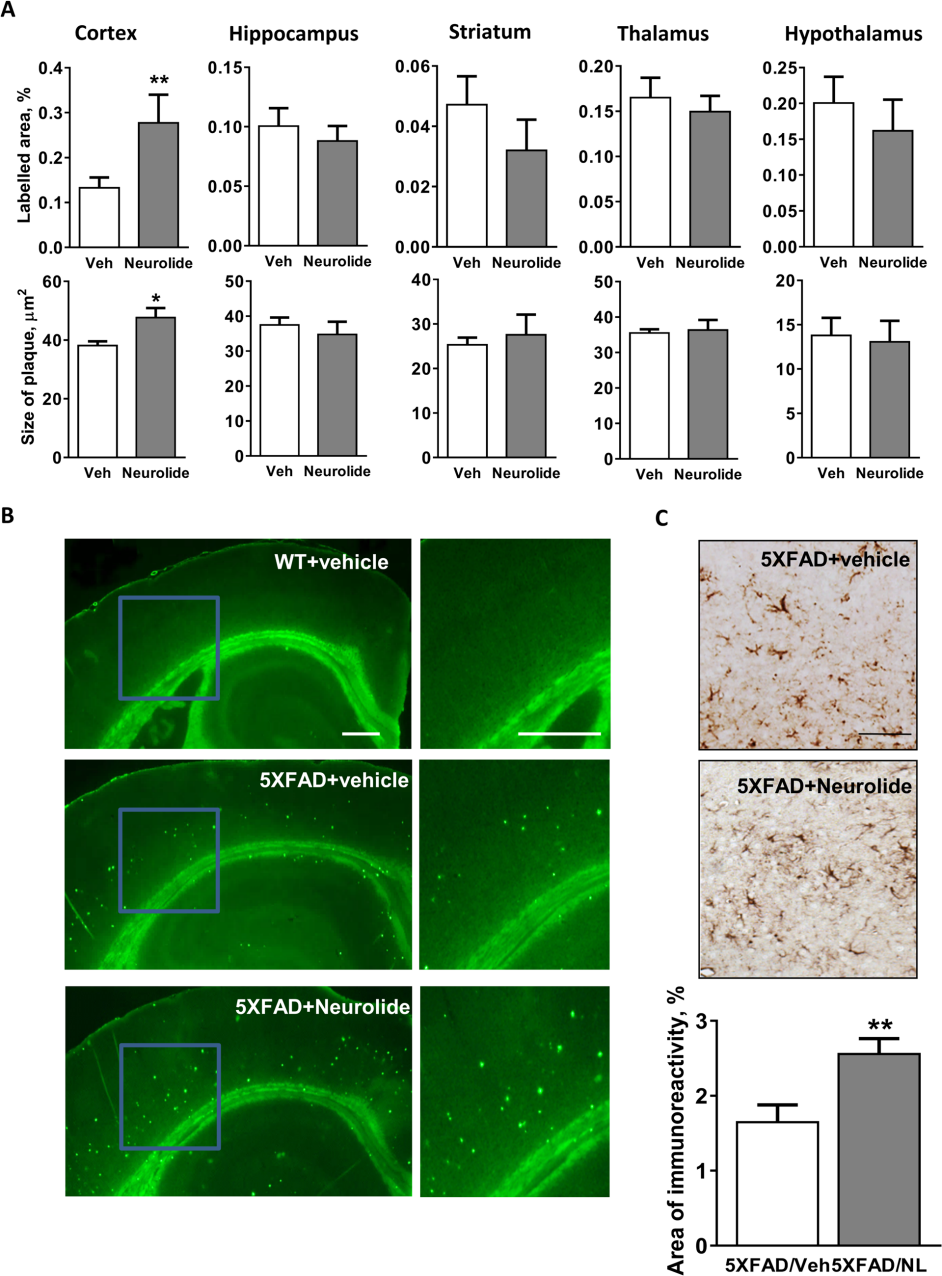
Region-dependent neurolide effect on plaque burden in the brain of 5XFAD mice. **A** The relative area of Aβ amyloid plaques (top row) and average plaque size (bottom row) measured in the cortex, hippocampus, striatum, thalamus, and hypothalamus of 5XFAD mice are depicted. Serial coronal sections of whole brain from 5XFAD mice treated with either vehicle (*n* = 13) or neurolide (*n* = 11) were stained with Thioflavine S, and the relative area (top row) and size of Aβ amyloid plaques (bottom row) were quantified using the ImageJ software. **B** Representative micrographs show Tioflavine S staining of brain sections from WT (top) and vehicle- (middle) and neurolide-treated (bottom) 5XFAD mice. Scale bar, 100 μm. **C** Quantification of GFAP-positive immunoreactivity in the cortex of 5XFAD mice after treatment with vehicle (*n* = 13) or neurolide (*n* = 11). Representative images of GFAP-positive immunoreactivity in cortex areas of vehicle- and neurolide-treated 5XFAD mice are depicted. Scale bar, 50 μm

5XFAD mice are known to show high Aβ plaque loads at 4–6 months of age [31]. To investigate whether neurolide treatment has an effect on the number and size of Aβ plaques, brains from 5-month-old 5XFAD mice and littermates controls, treated with either vehicle or neurolide, were subjected to thioflavin-S staining, which labels aggregated Aβ plaques in histological samples [31]. Five different brain regions, including cortex, hippocampus, striatum, thalamus, and hypothalamus, were analyzed for differences in the area covered by plaques and for the average size of the plaques. Compared with saline-treated 5XFAD mice, 5XFAD mice treated with neurolide showed a significant increase in the fractional area of plaques, as well as in the average size of thioflavin-S-positive plaques in the cortex (Fig. 4A, B). However, in other brain regions, no significant difference in either plaque area or plaque size was observed (Fig. 4 A, B). As expected, there were no plaques in either control or neurolide-treated WT brains (Fig. 4B), confirming that WT rodents do not develop this pathology [32]. This indicates that neurolide does not inhibit plaque formation in vivo but instead promotes plaque formation in the cortex.

The formation of Aβ plaques in 5XFAD mice is accompanied by astrogliosis [31]. Quantification of the area of GFAP-immunoreactivity also confirmed a significant increase in gliosis in the cortex area of neurolide-treated 5XFAD mice (*p* < 0.01, Fig. 4C).

### NEP Activity Is Inhibited by Both Neurolide and NL1

**Fig. 5:**
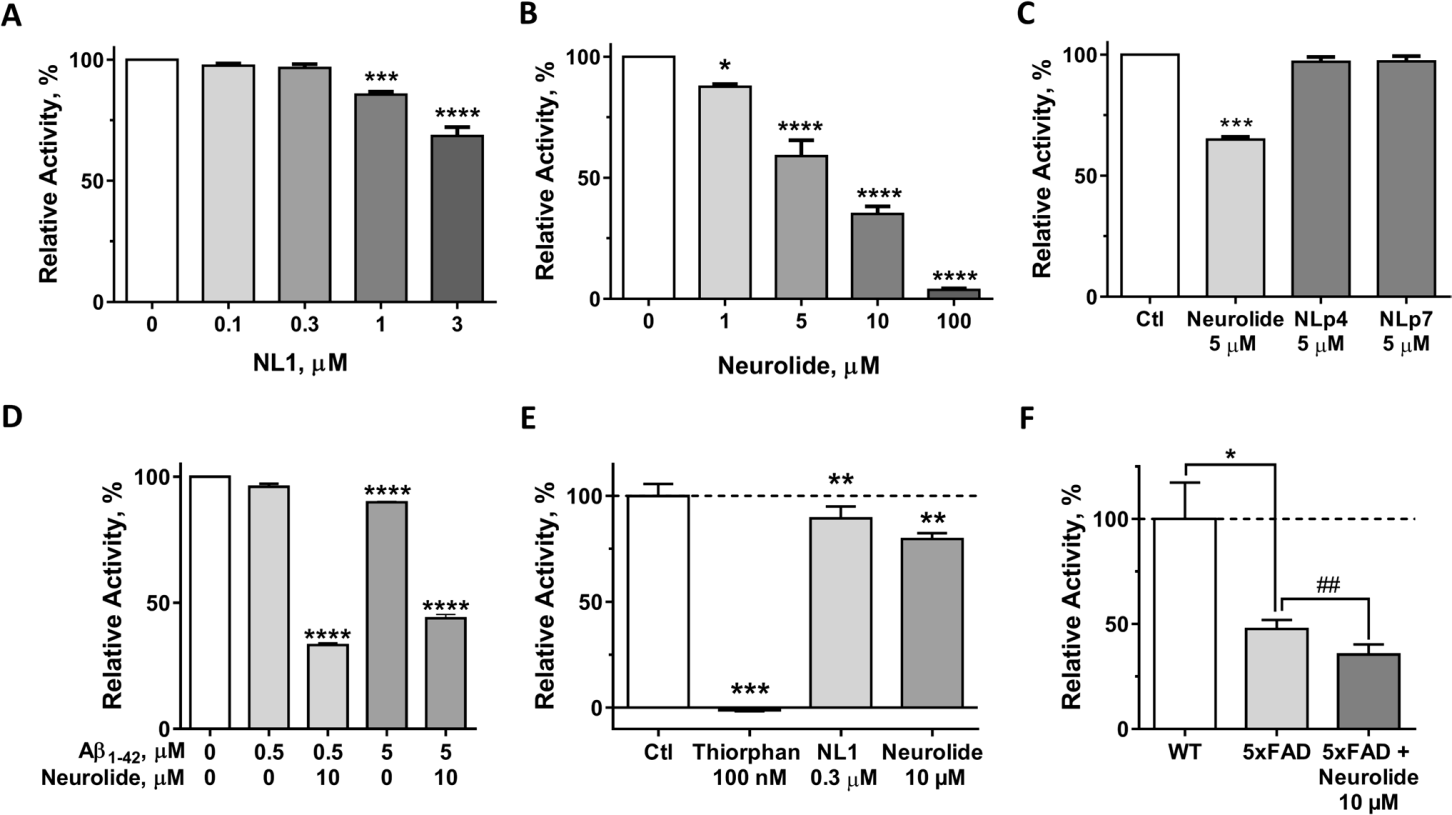
NL1 and neurolide effects on neprilysin activity. Both NL1 (**A**) and neurolide (**B**) decreased the cleavage activity of recombinant neprilysin in a dose-dependent manner. **C** Neurolide, but not other NL1-derived peptides, NLp4 and NLp7, down regulates the activity of recombinant neprilysin. **D** Neurolide (10 μM) downregulates the activity of recombinant neprilysin in the presence of Aβ_1-42_ applied either in 0.5 μM or 5 μM. At **A**–**D** panels data from four to five independent experiments are shown and compared with vehicle alone. **E** Both NL1 and neurolide downregulate the activity of neprilysin, extracted from brains of WT mice (*n* = 5). Treatment of brain extracts with Thiorphan (100 nM) confirm the neprilysin activity in prepared brain fractions and used as a negative control. **F** Activity of neprilysin extracted from brains of 5XFAD mice (*n* = 6) is significantly lower compared to WT mice (*n* = 5) and is further significantly attenuated by treatment with 10 μM neurolide (*n* = 6). In **E**–**F**, neprilysin activity in brain extracts was normalized to the activity of synthetic neprilysin in each experiment, set as 100% (dashed line). Values are mean ± SEM

In an attempt to explain the increase in plaque formation following the treatment with neurolide, we investigated the effect of NL1 and neurolide on neprilysin (NEP), a membrane-bound zinc-metalloendoprotease, which is primarily responsible for clearing Aβ from the brain. We employed an in vitro enzyme activity assay with recombinant NEP to test whether NL1 and/or neurolide might attenuate the activity of this enzyme. The results showed that both sNL1 and neurolide significantly inhibited the activity of recombinant NEP in a dose-dependent manner (Fig. 5A, B). The neurolide effect was sequence-specific because two other NL1-derived peptides, NLp4 and NLp7, applied in equimolar concentrations, had no effect on NEP activity (Fig. 5C). Furthermore, neurolide significantly inhibited NEP activity in vitro in the presence of different doses of Aβ peptide (Fig. 5D). We sought to further validate these effects with brain-derived NEP. For this purpose, NEP-enriched brain membrane fraction was extracted from the brain tissue of WT and 5XFAD mice (see Material and Methods for details) and tested for NEP activity. Incubation of WT mice NEP-enriched fractions with either sNL1 (0.3 μM) or neurolide (10 μM) significantly decreased the activity of brain-derived NEP (Fig. 5E), thus confirming that NL1 attenuates NEP activity, most likely via the neurolide motif. Co-incubation of brain-derived NEP with Thiorphan (100 nM), a known inhibitor for NEP, completely blocked the NEP activity, confirming that NEP in the membrane fractions was functional (Fig. 5E). Furthermore, NEP-enriched brain fractions derived from 5XFAD mice treated with 10 μM neurolide also showed significant decrease in NEP activity compared to untreated 5XFAD control (Fig. 5F). Of note, 5XFAD mice showed significantly less NEP activity compared to wild-type mice, confirming the previous observations (Fig. 5F) [6].

## Discussion

In the current study, we verified the interaction between NL1 and Aβ and further explored the potential involvement of NL1 in the Aβ-mediated development of AD pathology.

A number of studies have indicated that Aβ, particularly in the form of Aβ oligomeric peptides, targets the synaptic machinery, which might be a critical step responsible for the development of AD pathology [1–3, 33–35]. An antibody against Aβ oligomers showed accumulations as discrete puncta at postsynaptic terminals [36], suggesting the potential involvement of membrane-anchored Aβ-binding sites to attract oligomeric Aβ peptide to synapses [37, 38]. Thus, the identification of potential receptors responsible for Aβ amyloid binding within the synaptic structure is crucial. Previously, a number of synaptic proteins capable of binding to Aβ have been proposed, including Na + /K + ATPase alpha3 subunit (NKAα3), mGluR5, NMDA receptor, prion protein, Sigma-2 receptor/progesterone receptor membrane component 1 (PGRMC1), Freezled receptor, insulin receptor, and NL1 (reviewed in [1, 37]). Of note, the last protein, but not the closely related NL2, was proposed as a local synaptic nucleation factor for the Aβ peptide [21]. Moreover, direct interaction between sNL1 and Aβ at the postsynaptic membrane has been previously confirmed by coimmunoprecipitation and immunofluorescent staining [30]. Here, we confirmed the NL1-Aβ interaction and proposed that the peptide motif encompassing the splice site B of NL1, neurolide, represents the binding site for Aβ within the extracellular domain of NL1. Indeed, similarly to sNL1, neurolide binds to Aβ_1-40_ with nanomolar affinity and attenuates Aβ_1-42_ aggregation in vitro. However, the binding affinity of neurolide to Aβ was approximately 2 times lower than that of the parent sNL1 domain; therefore, other regions of the extracellular NL1 domain may be involved in the interaction with Aβ. Further binding experiments with mutated NL1 or NL1-specific splice site isoforms and consistent Aβ peptide oligomers would be required to confirm the insert B involvement in NL1-Aβ interaction. Notably, neurolide can bind to presynaptic ligand of NL1, NX1β [22], and Aβ also has been recently shown to interact with presynaptic NXs via the NL1-binding motif and, consequently, can reduce β-NXs expression [39, 40]. In addition, reduced level of hippocampal NL1 was recently observed in patients with AD [41]. Although the potential interference of the NXs-NL1 interaction by Aβ oligomers should be investigated in more detail on structural and functional levels, our present data may further explain the synaptic toxicity and memory impairment induced by the Aβ peptide.

The rapid accumulation of Aβ deposits in young adult 5XFAD mice has been shown to start in deep cortical layers and in the subiculum, accompanied by astrogliosis, and then spread to other brain regions [31]. Systemic treatment of 5XFAD mice with neurolide for 10 weeks (from 2 to ~ 5 months; Fig. 3A) overlapped with the most active steps of plaque formation specifically in the cortical area, where the peptide seemed to increase significantly the Aβ amyloid burden. This higher accumulation of Aβ plaques promoted by the neurolide treatment prompted us to look for an explanation for this phenomenon. A possible explanation is that exogenously applied neurolide, produced in tetrameric form, i.e., as four monomers coupled together [22], might function as a dual agonist for both synaptically located NX-1β and Aβ_1-42_ peptide, abundantly produced in 5XFAD transgenic mice, thus potentially providing additional anchor points for beta-amyloid nucleation. It is noteworthy that NL1 has been shown to have a dual effect on Aβ aggregation: at high concentrations (1 μM), sNL1 was able to inhibit Aβ aggregation, which is also confirmed by our results (Fig. 2); whereas at low concentration, such as 1 nM, NL1 accelerated the aggregation [21]. Systemically given neurolide displayed a plasma/CSF ratio of approximately 20, reaching only nanogram/low microgram levels in CSF within 30 min after single s.c. administration [23]. Although further research would be required to investigate neurolide concentration-dependent effects on Aβ aggregation, we speculate that neurolide might reach only low-level concentrations within brain parenchyma upon systemic treatment and, thus, similarly to low-level of sNL1, induce Aβ aggregation in brain tissues. Another explanation for neurolide-promoted plaque formation in 5XFAD mice could be the decrease in NEP activity induced by both neurolide and NL1. NEP is one of the key enzymes involved in the metabolism of a number of peptides including Aβ, which encompassed 11 NEP-specific cleavage sites [5, 42]. Consistently, a decrease of NEP activity was previously detected in brains of AD patients (reviewed in [5, 43]) and in 5XFAD mice brains [6]. Our results also confirmed the decreased activity level of NEP extracted from 5XFAD mice brains compared to WT. Thus, the attenuation of NEP activity by the neurolide peptide and by sNL1, observed here, would explain, at least in part, the increased accumulation of Aβ plaque-like formations within brain parenchyma of neurolide-treated 5XFAD mice. Further studies are required to elucidate the possible mechanism behind NL1- and neurolide-induced decrease of NEP activity, including potential direct NL1-NEP interaction.

In the present study, treatment with neurolide differentially modulated the cognitive behavior of healthy mice and mice with AD-like induced pathology. In particular, long-term treatment with neurolide did not enhance, or even decrease, memory in WT mice but improved working memory affected by 5XFAD mutations (Fig. 3D). Considering that neurolide attenuates NEP activity, which may be involved in memory formation under nonpathological conditions [44], we suggest that the reduced cognitive behavior observed in neurolide-treated WT mice might be partly explained by the decrease in NEP function. However, other reasons, such as the ability of neurolide to interact with synaptic NX1β, thereby potentially competing with membrane-bound NL1, might also contribute to memory decline in healthy mice. Interestingly, compared with vehicle-treated WT mice, vehicle-treated 5XFAD animals displayed a significant memory decline in the novel object recognition test, which is strongly linked to activity in the cortical area [45, 46], whereas no such difference was observed in the hippocampal-dependent Y-maze test (spontaneous alterations). These observations are in line with our histological data showing the accumulation of Aβ amyloid plaques and accompanied gliosis specifically in the cortex. Of note, the differential neurolide effect on behavior is consistent with previous studies, wherein neurolide had no effect on the spatial learning, social behavior, and locomotor activity of healthy rats [23, 47] but counteracted spatial memory deficits induced by injection with an NMDAR-antagonist, MK801 [23].

In summary, in the present study, we confirmed the ability of NL1 to bind to Aβ and identified a neurolide motif covering the B splice site of NL1 as an Aβ-binding site. Our findings show the differential effect of neurolide on the cognitive behavior of mice under physiological and AD-like pathological conditions. Moreover, for the first time, we showed that both sNL1 and neurolide can inhibit NEP activity, thus emphasizing its role in the development of AD pathology.

## Supplementary Information

Below is the link to the electronic supplementary material.

Supplementary Fig. 1. Binding of neurolide to the reverse version of Aβ (Aβ_40-1_). ELISA plates, pre-coated with recombinant NL1 (3.125 nM), were incubated for 4 h with biotinylated Aβ_40-1_ premixed with serially diluted neurolide, prepared in triplicates. The signal was developed using streptavidin HRP conjugate (1:1000) and the reaction was terminated with 0.2 M H_2_SO_4_. (PNG 42 kb)

High resolution image (TIF 561 kb)

Supplementary Fig. 2. List of NL1-derived peptide motifs showing similarity to the Aβ_25-35_ sequence. Sequence alignment was conducted using the freely accessible Clustal Omega package. Symbols used for alignment interpretation: *, conserved residues; :, residues with strongly similar properties; ., residues with weakly similar properties. (PNG 1512 kb)

High resolution image (TIF 6476 kb)

Supplementary Fig. 3. Both sNL1 and the NL1-derived peptide, neurolide, remained intact after the prolonged incubation under the aggregation assay conditions. NL1 (200 nM; R&D Systems) and biotinylated neurolide (6.7 μM; Schafer-N) were dissolved in the aggregation buffer, incubated at 37°C for 8 days, followed by conventional immunoblotting. Freshly prepared non-incubated samples were used as corresponding controls. Protein or peptide samples were transferred to PVDF membranes after separation by SDS-PAGE. The blots were blocked with 5% non-fat dry milk in PBST and probed with either anti-6x-His tag monoclonal antibody (1:2000) followed by secondary anti-mouse horseradish peroxidase (HRP)-conjugated secondary antibody (1:10,000) to detect sNL1 or streptavidin-HRP complex (1:5000) to detect b-neurolide. (PNG 98 kb)

High resolution image (TIF 1157 kb)

## Data Availability

No datasets were generated or analysed during the current study.

## Abbreviations

Aβ: Amyloid-β peptide
AD: Alzheimer’s disease
IDE: Insulin-degrading enzyme
NEP: Neprilysin
NL: Neuroligin
NMDA: N-methyl-d-aspartate
NX: Neurexin
PBS: Phosphate buffer saline
PDB: Protein data bank
RU: Resonance units
SPR: Surface plasmon resonance
