# Supplementary figures and images for: A Peptide Motif Covering Splice Site B in Neuroligin-1 Binds to Aβ and Acts as a Neprilysin Inhibitor
Source: Mol Neurobiol. 2024 Sep 11;62(3):3244–57. doi: 10.1007/s12035-024-04475-z (PMC11790763; doi:10.1007/s12035-024-04475-z)

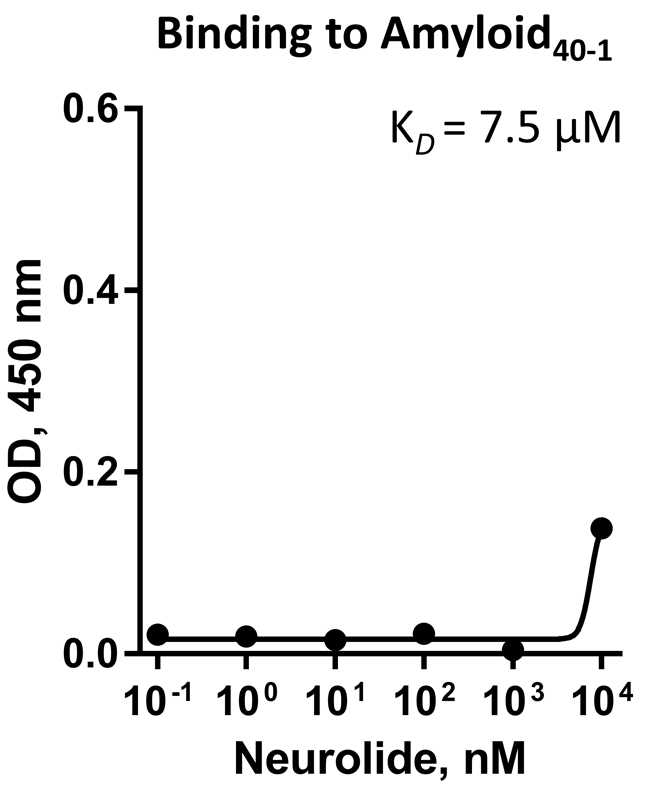

Supplement: Supplementary file 1 — Supplementary Fig. 1. Binding of neurolide to the reverse version of Aβ (Aβ40-1). ELISA plates, pre-coated with recombinant NL1 (3.125 nM), were incubated for 4 h with biotinylated Aβ40-1 premixed with serially diluted neurolide, prepared in triplicates. The signal was developed using streptavidin HRP conjugate (1:1000) and the reaction was terminated with 0.2 M H2SO4. (PNG 42 kb) [file 12035_2024_4475_Fig6_ESM.png]

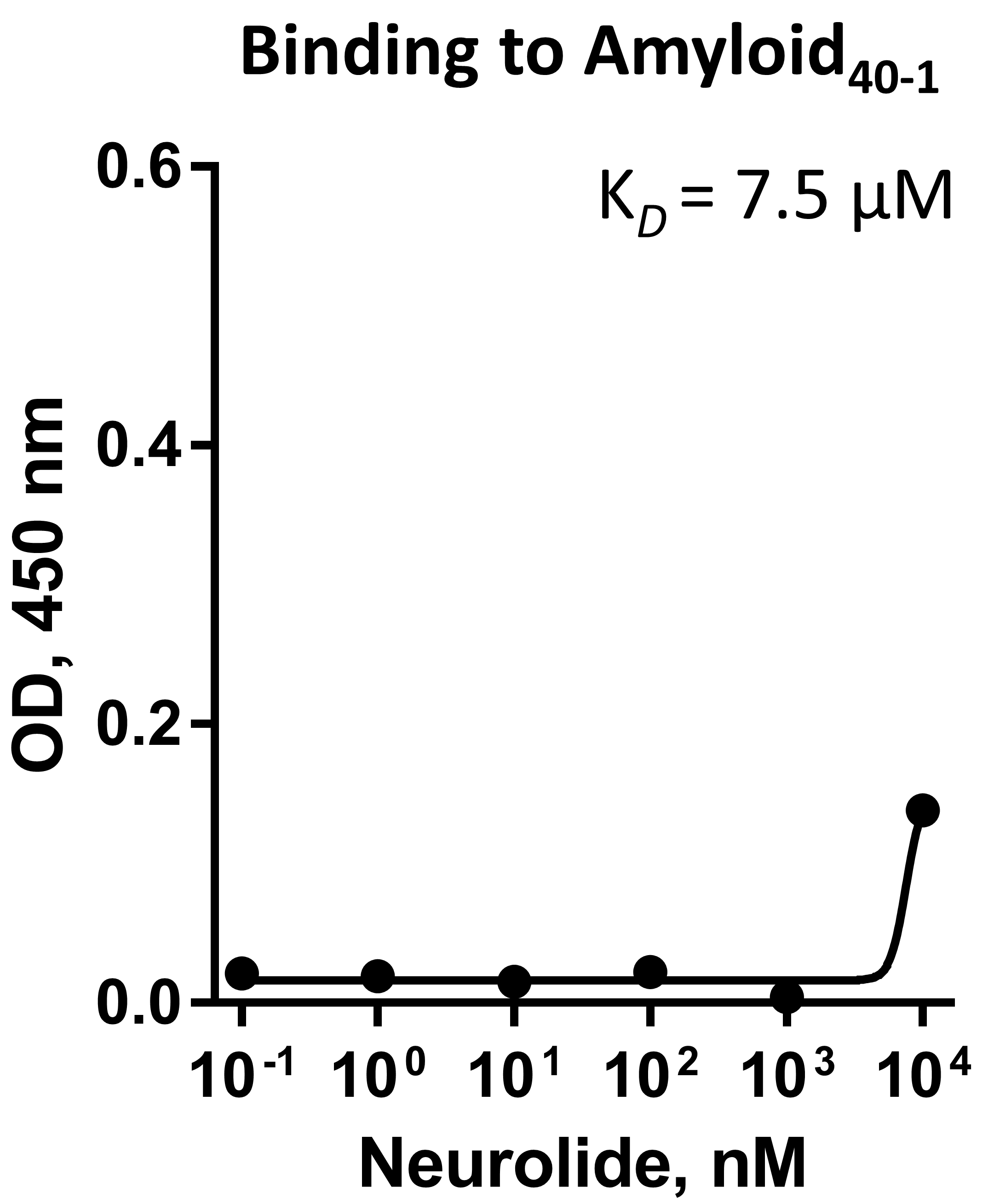

Supplement: Supplementary file 2 — High resolution image (TIF 561 kb) [file 12035_2024_4475_MOESM1_ESM.tif]

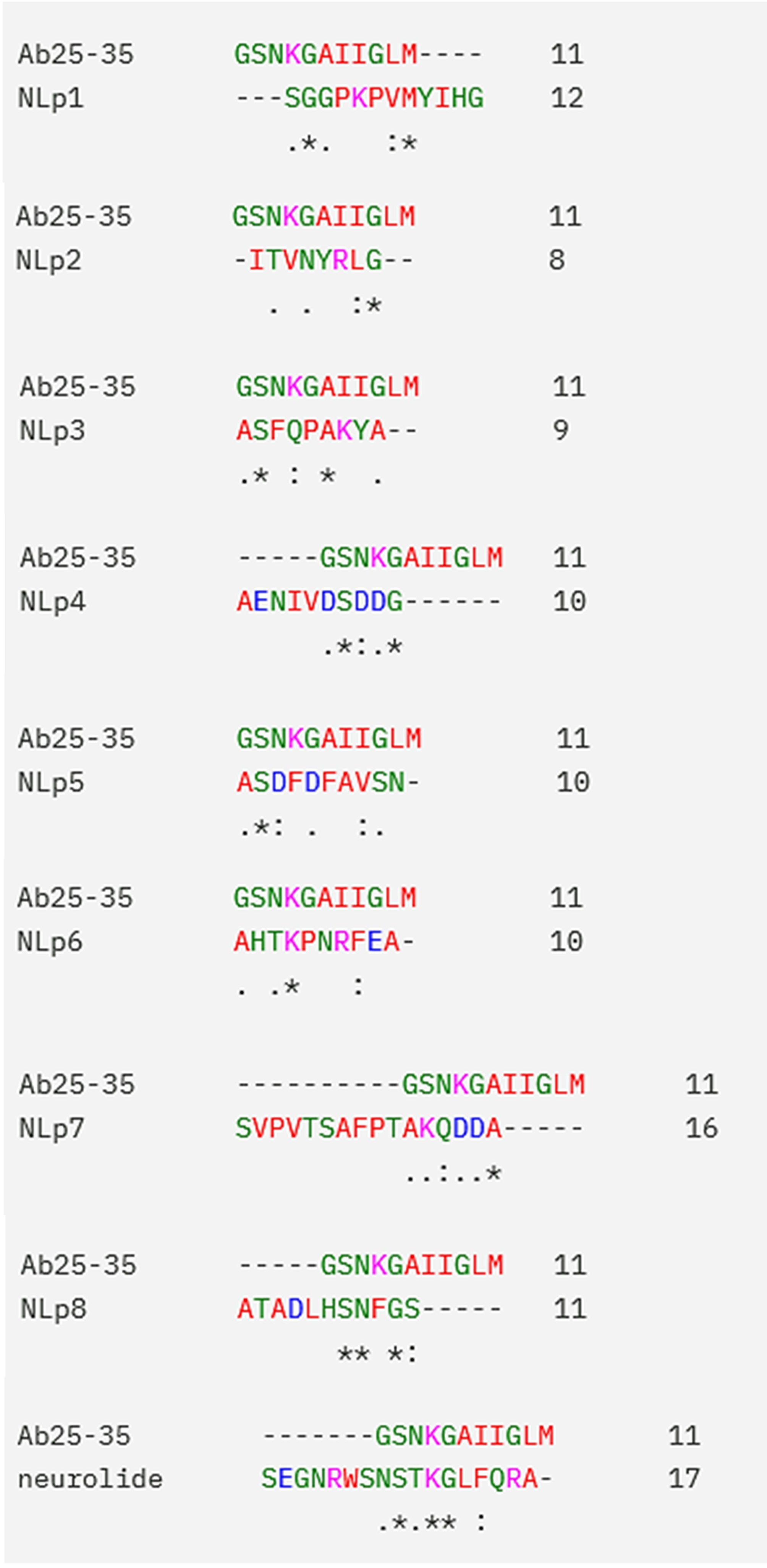

Supplement: Supplementary file 3 — Supplementary Fig. 2. List of NL1-derived peptide motifs showing similarity to the Aβ25-35 sequence. Sequence alignment was conducted using the freely accessible Clustal Omega package. Symbols used for alignment interpretation: *, conserved residues; :, residues with strongly similar properties; ., residues with weakly similar properties. (PNG 1512 kb) [file 12035_2024_4475_Fig7_ESM.png]

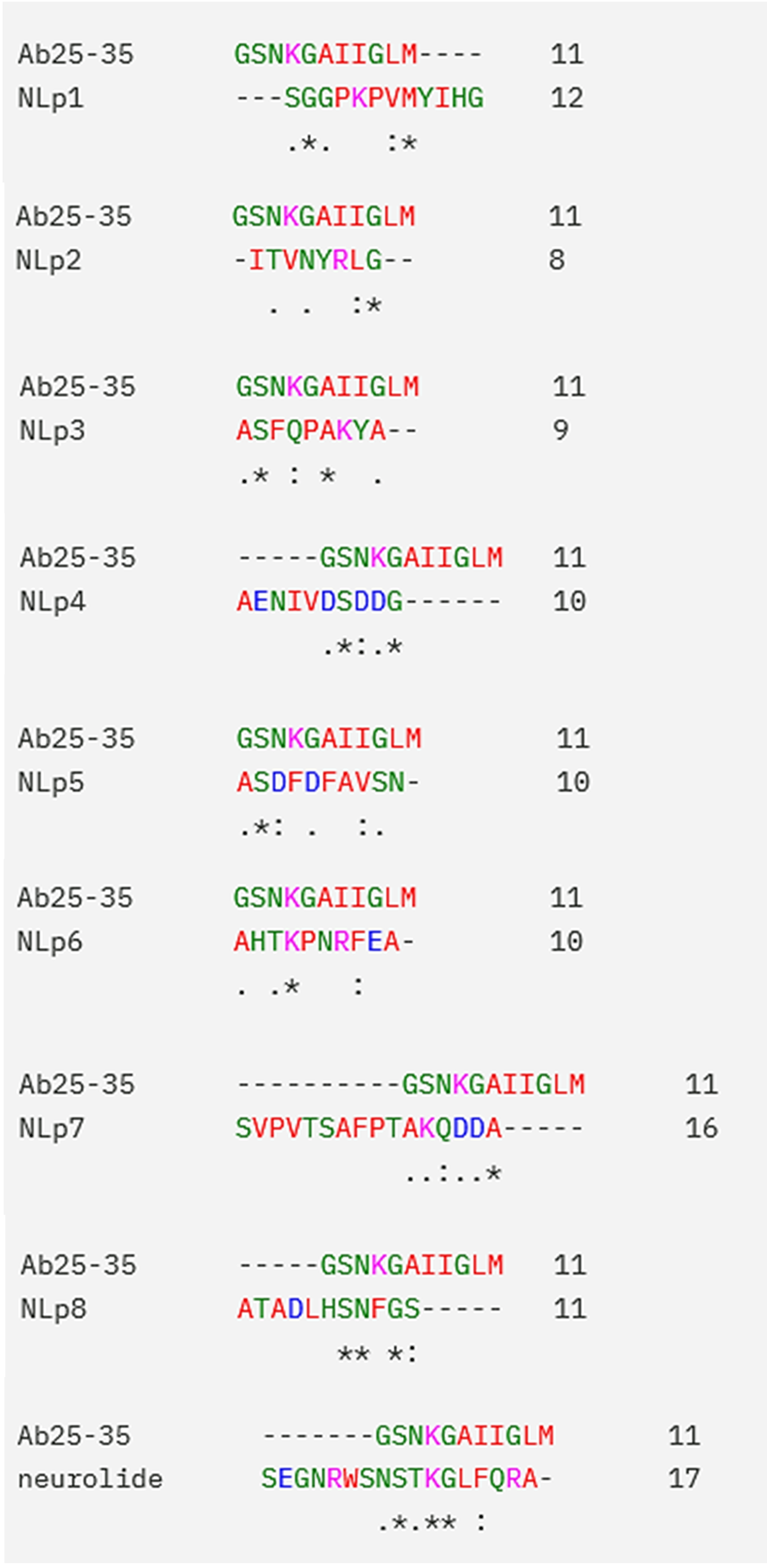

Supplement: Supplementary file 4 — High resolution image (TIF 6476 kb) [file 12035_2024_4475_MOESM2_ESM.tif]

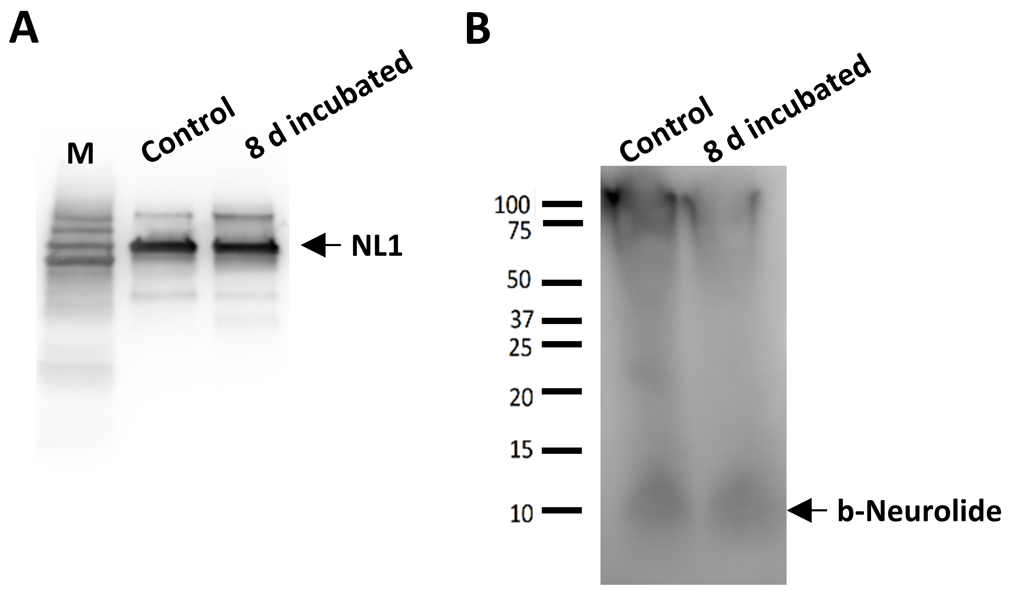

Supplement: Supplementary file 5 — Supplementary Fig. 3. Both sNL1 and the NL1-derived peptide, neurolide, remained intact after the prolonged incubation under the aggregation assay conditions. NL1 (200 nM; R&D Systems) and biotinylated neurolide (6.7 μM; Schafer-N) were dissolved in the aggregation buffer, incubated at 37°C for 8 days, followed by conventional immunoblotting. Freshly prepared non-incubated samples were used as corresponding controls. Protein or peptide samples were transferred to PVDF membranes after separation by SDS-PAGE. The blots were blocked with 5% non-fat dry milk in PBST and probed with either anti-6x-His tag monoclonal antibody (1:2000) followed by secondary anti-mouse horseradish peroxidase (HRP)-conjugated secondary antibody (1:10,000) to detect sNL1 or streptavidin-HRP complex (1:5000) to detect b-neurolide. (PNG 98 kb) [file 12035_2024_4475_Fig8_ESM.png]

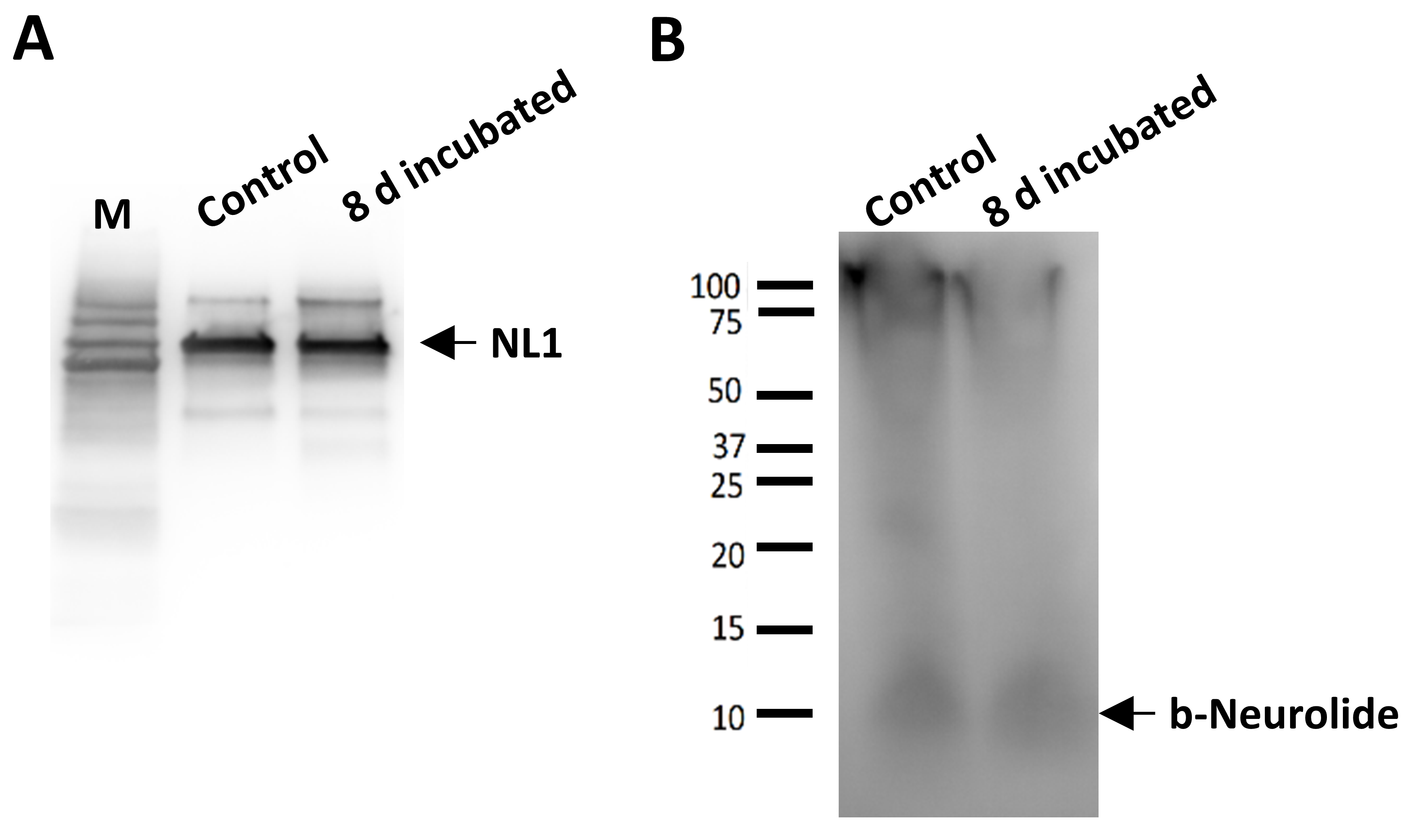

Supplement: Supplementary file 6 — High resolution image (TIF 1157 kb) [file 12035_2024_4475_MOESM3_ESM.tif]
